# Transition metal doped Sb@SnO_2_ nanoparticles for photochemical and electrochemical oxidation of cysteine

**DOI:** 10.1038/s41598-018-30962-0

**Published:** 2018-08-17

**Authors:** Yeonwoo Kim, Sena Yang, Yeji Kang, Byung-Kwon Kim, Hangil Lee

**Affiliations:** 1 Molecular-Level Interfaces Research Center, Department of Chemistry, KAIST, Daejeon, 34141 Republic of Korea; 2 Center for Nano Characterization, Korea Research Institute of Standards and Science, Daejeon, 305-400 Republic of Korea; 3 Department of Chemistry, Sookmyung Women’s University, Seoul, 04310 Republic of Korea

## Abstract

Transition metal-doped SnO_2_ nanoparticles (TM-SnO_2_) were synthesized by applying a thermos-synthesis method, which first involved doping SnO_2_ with Sb and then with transition metals (TM = Cr, Mn, Fe, or Co) of various concentrations to enhance a catalytic effect of SnO_2_. The doped particles were then analyzed by using various surface analysis techniques such as transmission electron microscopy (TEM), X-ray diffraction (XRD), scanning transmission X-ray microscopy (STXM), and high-resolution photoemission spectroscopy (HRPES). We evaluated the catalytic effects of these doped particles on the oxidation of *L*-cysteine (Cys) in aqueous solution by taking electrochemical measurements and on the photocatalytic oxidation of Cys by using HRPES under UV illumination. Through the spectral analysis, we found that the Cr- and Mn-doped SnO_2_ nanoparticles exhibit enhanced catalytic activities, which according to the various surface analyses were due to the effects of the sizes of the particles and electronegativity differences between the dopant metal and SnO_2_.

## Introduction

SnO_2_ is well known as one of the best smart materials and is used in an impressive range of applications including solar cells^1–4^, catalyst supports^5–8^, and chemical sensors^9–12^. It has been particularly well regarded as a highly efficient catalyst in many gas conversions such as those involving CO, NO_2_, and some hydrocarbons^13,14^. Mechanistic studies of catalytic reactions using SnO_2_ have been researched in several ways. It is, however, still necessary to enhance the catalytic effects of SnO_2_, and attempts at such enhancements have been carried by inserting metals or anions, but such projects need to be mindful of the costs of the fabricated catalyst as well as its efficiency^15–17^. Doping SnO_2_ with Sb (to produce Sb@SnO_2_) is one of the best-known modifications of the SnO_2_ catalyst, and here the Sn 5*s* state was shown to contribute to the electrical conductivity because this state is related to the catalytic activity^18–20^.

We have been pursuing a strategy of additional doping Sb@SnO_2_ nanoparticles with transition metals to further enhance the catalytic activity of these nanoparticles. The procedure described below has in many cases been shown to increase their catalytic performance significantly. Transition metals such as Cr and Fe are the most feasible dopant candidates because they possess various oxidation states and thus contribute to the enhancement of the catalytic activities. For this purpose, in the current study, we inserted various transition metal ions (TM^+^) into Sb@SnO_2_ nanoparticles, and then systematically compared the catalytic activities of these metal-doped Sb@SnO_2_ nanoparticles (TM-SnO_2_).

In this work, the effects of doped transition metals, in particular Cr, Mn, Fe, and Co, on catalytic properties of the Sb@SnO_2_ nanoparticles were studied when these metals were partially substituted into the nanoparticles. A one-pot synthesis was carried out to form uniformly sized (5~9 nm) catalyst nanoparticles and to maximize the 1 monolayer of the electron depletion layer. In particular, by commencing with TM-SnO_2_ nanoparticles, we successfully fabricated them through a thermos-synthesis method and then assessed their catalytic capacities by oxidizing *L*-cysteine (Cys) under ultra-high vacuum (UHV) conditions with 365-nm-wavelength UV light illumination by using high-resolution photoemission spectroscopy (HRPES), and in the solution phase by using electrochemistry. These reactions and analyses were also performed to determine the mechanism of the catalytic oxidation reaction. In addition, we also assessed the rate of the conversion of CO to CO_2_ by using a mass spectrometry.

Cys, a small amino acid with a thiol group (-SH), plays an important role in cellular homeostasis, in the biological activity of proteins, and in metabolism^21,22^. Abnormal levels of Cys lead to many diseases. For example, low levels of Cys give rise to liver damage, and to slow growth in children, whereas high levels of Cys have been associated with neurotoxicity^23^. Therefore, the detection of Cys levels is important in the fields of biology and diagnostics. Cys can be detected by taking electrochemical measurements and oxidation reaction^24–26^. Here, we used a catalytic oxidation and an electrochemical oxidation of Cys to demonstrate the possible application of synthesized TM-SnO_2_ as a catalyst for the sensitive detection of Cys.

We precisely compared the morphologies and electronic properties of the Sb@SnO_2_ nanoparticles doped with each of the four metals (i.e., Cr, Mn, Fe, or Co) by using transmission electron microscopy (TEM), X-ray diffraction (XRD), scanning transmission X-ray microscopy (STXM), and also assessed their catalytic activities by using high-resolution photoemission spectroscopy (HRPES), and electrochemical (EC) measurements. Through the spectral analyses, we found the catalytic properties of Cr-Sb@SnO_2_ (Cr-SnO_2_) and Mn-Sb@SnO_2_ (Mn-SnO_2_) to be enhanced over those of the original Sb@SnO_2_ nanoparticles and the other transition metal-doped Sb@SnO_2_ nanoparticles (i.e., Fe-Sb@SnO_2_ (Fe-SnO_2_) and Co-Sb@SnO_2_ (Co-SnO_2_)).

## Results and Discussion

### Synthesis

Sb@SnO_2_ nanoparticles were prepared by the thermal synthesis method as described below. Sn:Sb ratio was selected as 100:5 to maximize the electrical conductivity which helps not only applying the electrical conductivity in SEM, HRPES measurements but also increasing the catalytic performance by shallowing the conduction band minimum^27^. Around 5 mol% of Sb into the SnO_2_ matrix, Sb@SnO_2_ shows the lowest resistivity by n-type doping^28^ which was accomplished by the major contribution of Sn^5+^ state below 22 mol% of Sb atom. XPS data in previous study shows no Sb^3+^ state at 3.8 mol% which means the hole transport effect can be excluded in those ratio^29^. This structural design can be achieved the understanding of the only catalytic effects by electrons by fixing the major carrier component. That range of mole percent can also achieve UV absorption to confirm the photocatalytic activity. When Sb concentration is increased above 2 mol%, band gap of SnO_2_ is reduced from 3.95 eV to below 3.65 eV which can actively absorb the UV wavelength^27^.

### TEM and XRD measurements

**Figure 1:**
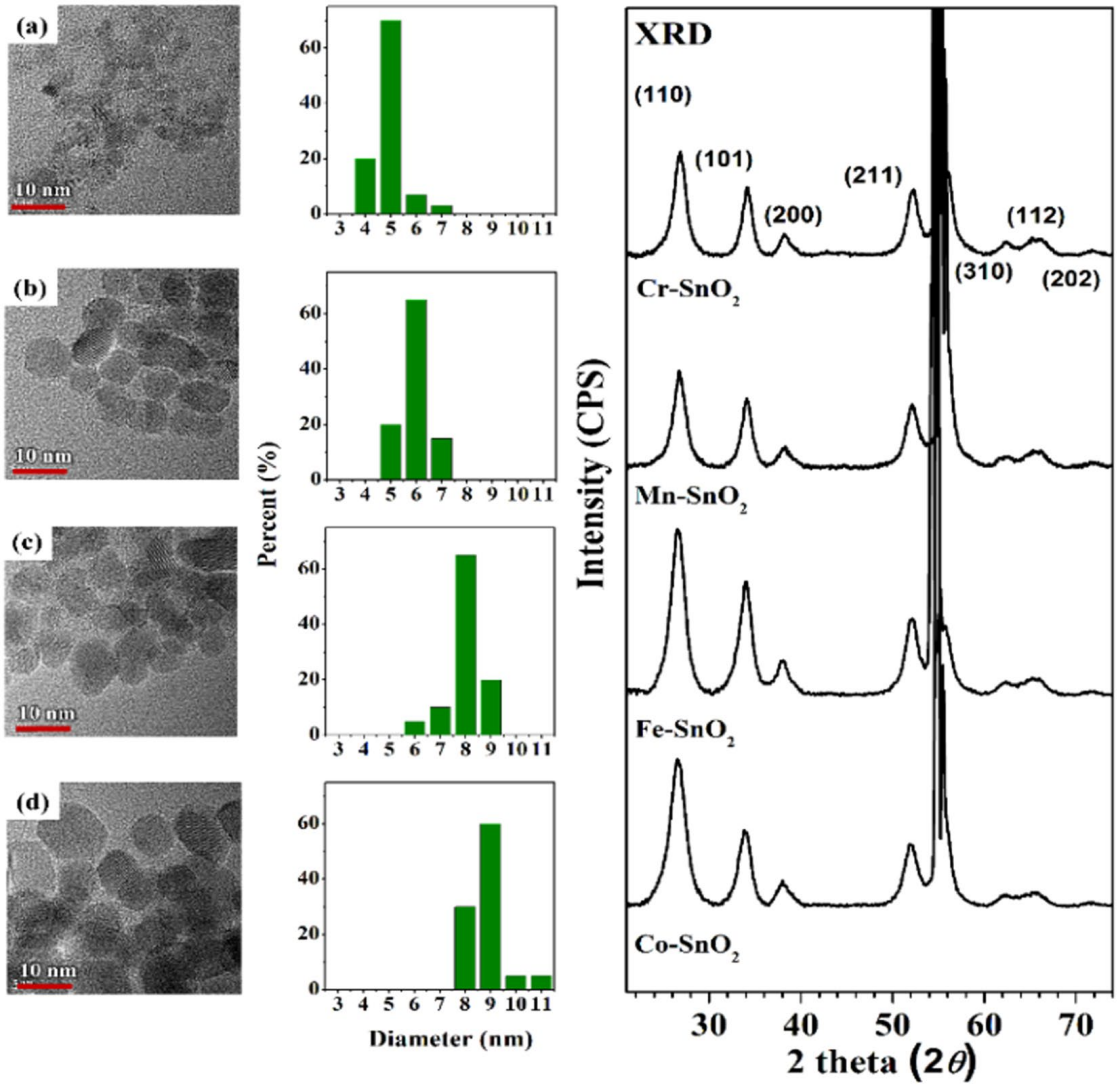
(Left) TEM images of the monodispersed 5 mole % TM-SnO_2_ particles, (middle) their size distributions, and (right) their XRD spectra, for (**a**) Cr, (**b**) Mn, (**c**) Fe, and (**d**) Co.

TEM images (Fig. 1) of 5 mole % TM-SnO_2_ nanoparticles were obtained in order to determine the size distributions and shapes of the particles. These images revealed these particles to have very fine structures with dimensions mostly between 5 and 9 nm. All of the diffraction peaks of their XRD spectra were assigned to SnO_2_ (cassiterite) since they were found to be consistent with the peaks at 2*θ = *26.8°, 34.1°, 38.2°, 52.0°, 62.2°, 65.2°, and 71.6° corresponding to the (110), (101), (200), (211), (310), (112), and (202) reflections (JCPDS card No. 41–1455). The TEM images and XRD patterns showed the formation of single-phase SnO_2_, indicative of successful doping of the transition metal into the SnO_2_ nanoparticles without any phase separation and segregation. Sharp 2*θ* = 55.88 is correspond to the Si substrate which was mentioned in method section.

### STXM measurements

**Figure 2:**
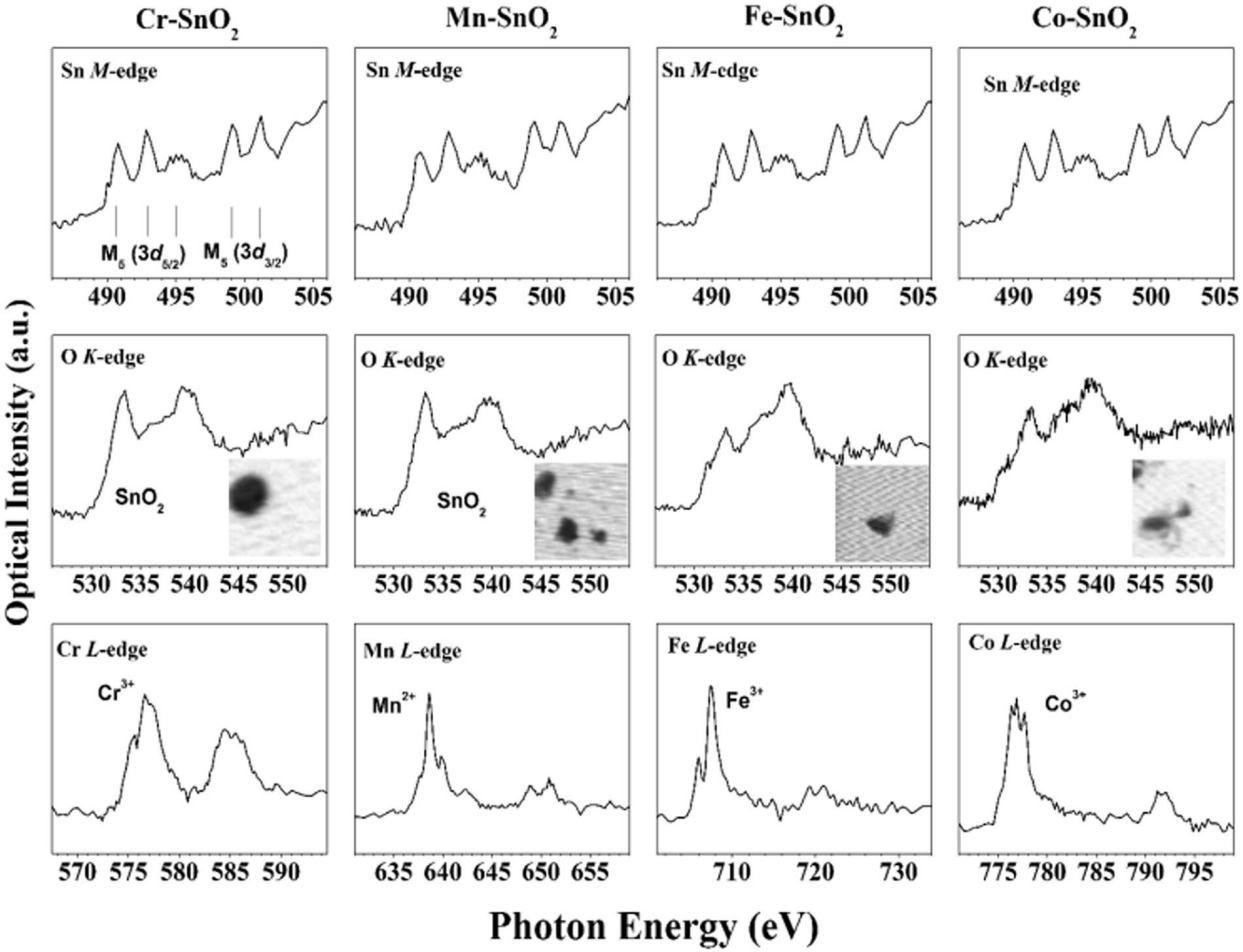
XAS spectra of (top panels) Sn *M*_4,5_-edges, (middle panels) O *K*-edge, and (bottom panels) doped transition metal *L*_2,3_-edge, and (middle panels) the corresponding stacked images, for 5 mole% Cr-SnO_2_, Mn-SnO_2_, Fe-SnO_2_, and Co-SnO_2_ nanoparticles.

Figure 2 shows the X-ray absorption spectra (XAS) and the corresponding stacked images (black regions of each inserted image) of the TM-SnO_2_. Three small features between 490 and 497 eV and two small features between 498 and 502 eV were observed in the spectra at the Sn *M*_4,5_-edges, which indicated the transition from the Sn^4+^ 3*d* state to the unoccupied *p* state. The features between 490 and 497 eV, and those between 498 and 502 eV, derived from Sn *M*_5_ (3*d*_5/2_ and 3*d*_3/2_), respectively^30,31^. The Sn *M*-edge measurements of all four TM-SnO_2_ were very similar.

On the other hand, the O *K*-edge spectra region showed a quite different tendency. As shown in O *K*-edges region, peaks were observed at 533 eV and 536 to 540 eV, and were due to the transition from the O 1 *s* state to the unoccupied *p* state, and from the O 2*p* state to the O 2*p* – Sn 5*p* hybrid orbital state, respectively. The shapes and intensities of the O *K*-edge peaks for Cr-SnO_2_ were very similar to those for Mn-SnO_2_ and to those for undoped SnO_2_^32,33^. However, the O *K*-edges of Fe-SnO_2_ and Co-SnO_2_ indicated more of the hybrid orbital (536 to 540 eV) than of the bare O 2*p* transition (533 eV), which may have been due to the Fe and Co dopants. The orbitals of these dopants each hybridized with the O 2*p* orbital according to the spectra. The oxidation states of the transition metal dopants were determined by the metal *L*-edges. For Cr-SnO_2_, peaks were observed at 576.0 eV and 577.0 eV with a shoulder at 578.4 eV, corresponding to the Cr^3+^ *L*_3_-edge^34^. For Mn-SnO_2_, a sharp peak was observed at 639.2 eV with a small feature at 640.7 eV, corresponding to the Mn^2+^ *L*_3_-edge^35^. For Fe-SnO_2_, a sharp peak was observed at 708.5 eV with a small peak at 706.6 eV, corresponding to the Fe^3+^ *L*_3_-edge^36^. For Co-SnO_2_, a doublet was observed at 776.8 eV and 777.6 eV and was clearly separated from another doublet at 791.2 eV and 792.0 eV, and these peaks were confirmed as Co^3+^ *L*_3_ and *L*_2_-edges, respectively^34^. These results indicated that STXM measurements can distinguish Cr-SnO_2_ and Mn-SnO_2_ from Fe-SnO_2_ and Co-SnO_2_.

### Electrochemical redox reaction in the aqueous phase

**Figure 3:**
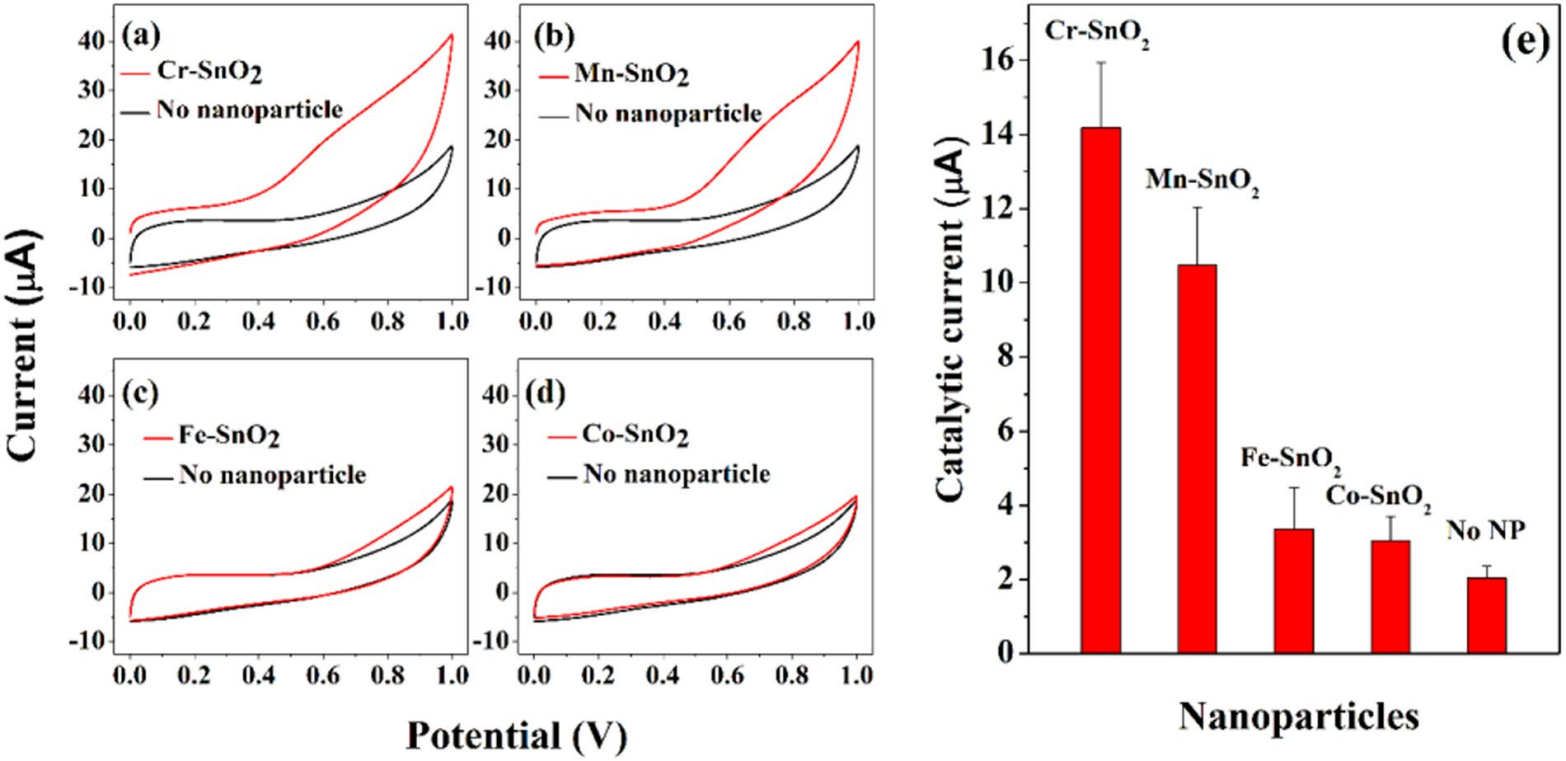
(**a**–**d**) CVs (at a scan rate of 50 mV/s) in PBS containing 10 mM Cys at a bare GCE (black lines) or GCEs modified (red lines) with 5 mole% (**a**) Cr-SnO_2_, (**b**) Mn-SnO_2_, (**c**) Fe-SnO_2_, and (**d**) Co-SnO_2_ nanoparticles. (**e**) Catalytic currents resulting from the electrochemical oxidation of Cys for the various types of TM-SnO_2_ nanoparticles.

CVs were obtained in a PBS solution containing 10 mM Cys at various types of GCEs irradiated by 365-nm-wavelength UV light. As shown in Fig. S1, a sluggish oxidation current was observed at a bare GCE because of the intrinsically slow electrochemical oxidation of Cys. To increase the current associated with the oxidation of Cys, GCEs modified with the TM-SnO_2_-Nafion catalysts were fabricated and tested, with the results shown in Fig. 3. The currents associated with the oxidation of Cys were 14.2 (±1.7) and 10.5 (±1.6) μA when using the GCEs modified with the Cr-SnO_2_ and Mn-SnO_2_, respectively — significantly greater (i.e., 7.1 and 5.2 times greater) than the 2.0 μA value observed when using only the bare GCE (Fig. 3e). In contrast, the currents generated when using the Fe-SnO_2_ and Co-SnO_2_ were only 3.4 (±1.1), and 3.1 (±0.6) μA, respectively, which were slightly (1.7 and 1.5 times) but not significantly greater than that for the bare GCE. These results revealed the importance of the type of metal doped into the SnO_2_ nanoparticles for catalyzing oxidation reactions, even when using small amounts (5%) of the doped metal, and specifically indicated the Cr-SnO_2_ and Mn-SnO_2_ to be good catalysts for the oxidation of Cys. Further investigations involving the optimization of the conditions are needed to selectively and sensitively detect Cys using Cr-SnO_2_ and Mn-SnO_2_ nanoparticles.

### Photocatalytic oxidation of *L*-cysteine

**Figure 4:**
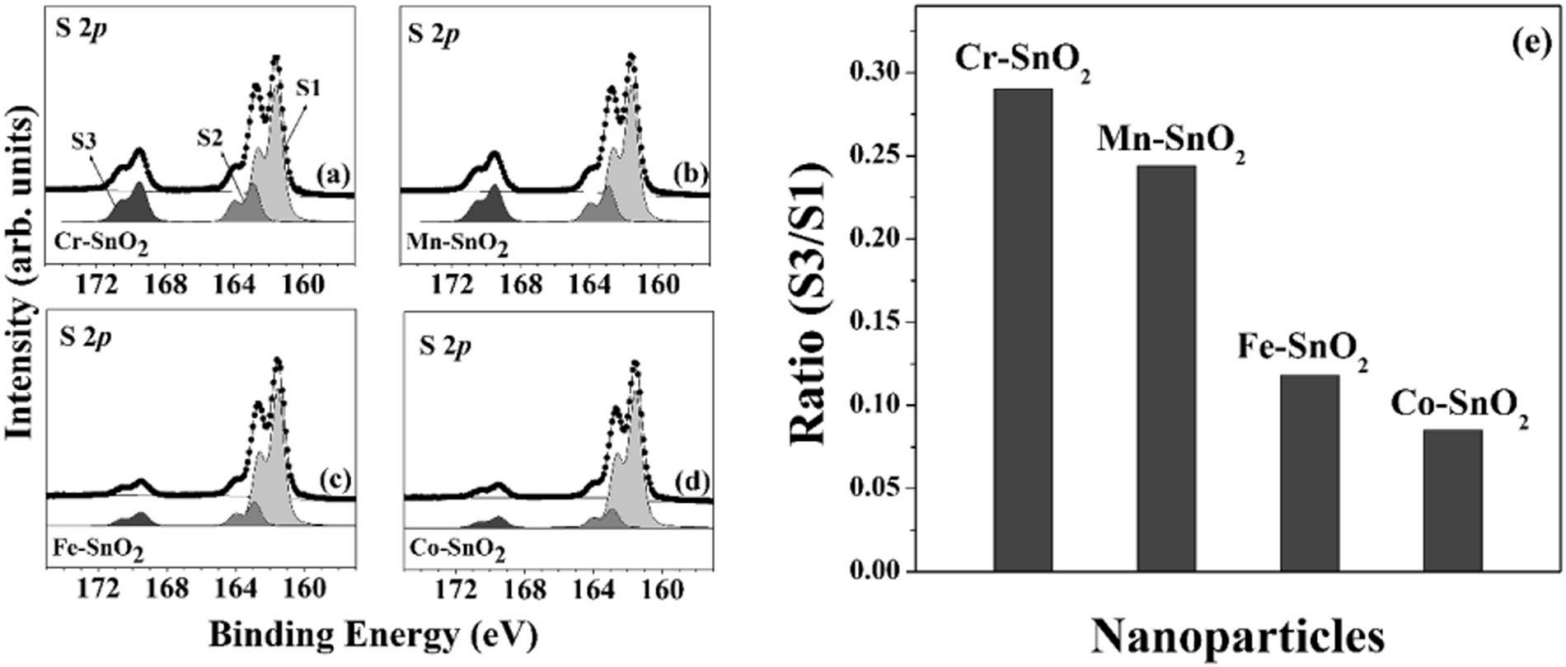
HRPES S 2*p* core-level spectra of the products of the photocatalytic oxidations of Cys (a 180 L solution) carried out in the presence of 5 mole% (**a**) Cr-SnO_2_, (**b**) Mn-SnO_2_, (**c**) Fe-SnO_2_, and (**d**) Co-SnO_2_ nanoparticles. (**e**) Values of the S3 to S1 ratio (see text), the four types of TM-SnO_2_ nanoparticles, resulting from 180 L exposure of Cys solutions to 365-nm-wavelength UV light, in order to assess the photocatalytic activity of each type of nanoparticle towards the oxidation of cysteine.

We determined the catalytic activities of the TM-SnO_2_ for the oxidation of Cys molecules. The surface-sensitive S 2*p* core-level HRPES spectra were acquired from the products of the exposure of a 180 L of Cys to the actual amount of oxygen used and 365-nm-wavelength UV light in the presence of each type of TM-SnO_2_ (Fig. 4(a–d)). As shown in these figures, three distinct 2*p*_3/2_ peaks were observed, at 161.5, 162.9, and 168.6 eV, which corresponded to the thiol group (-SH; denoted as S1), the bound state (denoted as S2), and sulfonic acid (SO_3_H) (denoted as S3), respectively. Since sulfonic acid has been shown to be an oxidation product of the thiol group^37,38^, we monitored the oxidation of Cys by measuring the ratio of the intensity of S1 to that of S3 for each of the four types of TM-SnO_2_. The results of these measurements (Fig. 4(a–d)) confirmed Cr-SnO_2_ and Mn-SnO_2_ to be effective catalysts. To determine the effects of metal doping on the catalytic performance, the ratio of S3 to S1 was determined for 5% mole dopant concentrations (Fig. 4(e)). In particular, Cr-SnO_2_ and Mn-SnO_2_ exhibited clear enhancements in photocatalytic activity, and these results were closely correlated with the EC results. We also performed experiments involving the conversion of CO to CO_2_ (Fig. S2) as described in Supplementary Information text to clarify the trend of the catalytic activities for these TM-SnO_2_ at various TM doping concentrations.

Through the characterizations of the electronic structures and of catalytic oxidation reactions for the four TM-SnO_2_, we found that two quite different catalytic measurements — the rate of electrochemical oxidation of Cys in aqueous solution and the rate of catalytic oxidation in ultra-high vacuum conditions — showed the same trends. In both cases, the Cr-SnO_2_ and Mn-SnO_2_ showed the highest and second highest catalytic activities, respectively. These same trends were found despite the measurements conditions (in aqueous and under UHV condition) being so different. These results suggest the effect of the identity of the doped metal on the catalytic activity of SnO_2_ to be independent of the environmental conditions.

Previous results about TM-SnO_2_ reported SnO_2_ nanoparticles doped with only one type of metal and reported only a single measurement phase (in aqueous or under UHV condition). Therefore, usual concepts were used to explain observed increases in catalytic activity upon metal doping. For example, for catalytic oxidation experiments carried out in vacuum conditions, band gap theory was frequently used to explain the catalytic activities of the doped TM-SnO_2_ due to the bandgap narrowing from the 3.6 eV value for the bare SnO_2_ nanoparticles upon being doped^39,40^. In general, the bandgap narrowing of a TM-SnO_2_ leads to enhanced photocatalytic activity. However, it showed slightly enhanced catalytic activities upon being doped with a metal. This explanation for the increase in the catalytic activity is well established in the field of photocatalysis. In contrast, increases in the electrochemical catalytic activities in the aqueous phase resulting from doping a catalyst with a metal have been explained by the doping causing an increase in the conductivity. Doping Fe into SnO_2_, for example, has been shown to increase its conductivity, and this increase in conductivity has been offered as an explanation for the observed increase in catalytic activity^41^. When analyzing the results of each individual experimental condition (aqueous condition or under vacuum) on its own, each corresponding explanation (narrowing the bandgap or increasing the conductivity) appears persuasive. However, in our experiments, the results showed the same trends for both conditions, and hence another explanation is needed for why doping causes an increase in catalytic activity.

One possible alternative explanation is the difference of the electronegativity between the doped transition metals and SnO_2_. The electronegativities (Allen scale) of Cr, Mn, Fe, and Co are 1.56, 1.60, 1.64, and 1.70 respectively^42,43^, while those of Sn and O are 1.72 and 3.52, respectively. When these transition metals, which have lower electronegativity values than Sn, are doped into the SnO_2_, hot spots for the oxidation of Cys are generated. Cys has electron-rich thiol (-SH) and amine (-NH_2_) groups that bind more strongly to these hot spots, which are positively charged, than to adjacent atoms. These hot spots are fairly attractive for the oxidation of the Cys.

Another reasonable explanation is that according to the O *K*-edge XAS region shown in Fig. 2, a higher proportion of less hybridized oxygen states (533 eV) appeared in Cr-SnO_2_ and Mn-SnO_2_ than in the other TM-SnO_2_. Those transition of the doped metal 3*d* to the O 2*p* unoccupied state can facilitate the removal of oxygen atoms from the SnO_2_ nanoparticles and promote the catalytic oxidation of Cys because oxygen vacancy site of SnO_2_ is an active site. According to the HRPES data of TM-SnO_2_ by increment of the amount of the dopants (Figs S3–S6), only Cr-SnO_2_ (Fig. S3) and Co-SnO_2_ (Fig. S6) show the additional state in valence band region. However, there have no relationship between the new states and catalytic performance which means the reaction did not occur by the hole transfer. The reasonable interpretation of binding sites and reactive charge is that the hot electrons are transferred to the oxygen vacancy sites (electron trap sites) to enhance the photo- and electro-chemical activities. In Figs S3–S6, there have 531.9 eV in O 1 *s* spectra which are assigned as oxygen vacancy sites^44^. The starting point of those materials are SnO_2_ with 5 mol% of Sb doping. This structural design has the electron-enhanced structure by the 100% Sb^5+^ doping (n-type SnO_2_). In addition to this structural design, electronegativity model supports the high catalytic performance by introduction of additional transition metals. In the cases of Fe-SnO_2_ and Co-SnO_2_, the electronegativities of Fe and Co are only slightly lower than that of Sn, and the proportion of O 2*p* orbitals that are hybridized with Sn 5*d* orbitals is larger, and tightly bound to the SnO_2_ nanoparticle. Correspondingly, doping the SnO_2_ nanoparticle with either Fe or Co yielded a smaller increase in the catalytic activities for Cys oxidation than did doping with Cr or Mn. Hence, we believe that this electronegativity hypothesis and less hybridization result between the doped metal 3*d* to the O 2*p* are fairly straightforward and reasonable, but requires further experiments to be proven.

## Conclusion

The results of catalytic oxidation through the EC measurements and the HRPES analysis, which showed the catalytic activities of Cr-SnO_2_ and Mn-SnO_2_ to be superior to those of the other metal-doped nanoparticles or bare SnO_2_ nanoparticles. It is in good agreement with our STXM measurements indicated the doped structures of Cr-SnO_2_ and Mn-SnO_2_ to be better arranged than those of SnO_2_, Fe-SnO_2_, and Co-SnO_2_. As a result, Cr-SnO_2_ and Mn-SnO_2_ showed higher catalytic activities due to the electronegativity differences and less hybridizations between the dopant metals and SnO_2_, which are induced by increasing the oxygen vacancy site of SnO_2_.

## Methods

### Preparation of precursor solutions

We prepared the precursor solutions by carrying out one-pot syntheses. 10 mmol of tin (IV) chloride pentahydrate (SnCl_4_∙5H_2_O, 98%) and 0.5 mmol of antimony (III) chloride (SbCl_3_, >99.0%) were added to 10 ml of 2-methoxy ethanol (≥99.9%). Dopants (M) were added in the form of the transition metal nitrate *n*-hydrate (TM(NO_3_)_*x*_∙*n*H_2_O) in desired amounts that were determined by calculating the mole fraction of the metal dopant according to the equation (moles of TM)/((moles of TM) + (moles of Sn)). Specifically, Cr(NO_3_)_3_·9H_2_O (99%), Mn(NO_3_)_2_·*x*H_2_O (98%), Fe(NO_3_)_3_·9H_2_O (98%), and Co(NO_3_)_2_·6H_2_O (≥98%) were used as dopants. All substances were purchased from Sigma Aldrich. Precursor solutions were stirred for 10 minutes. *L*-cysteine (Sigma Aldrich, 97% purity), and Nafion (Sigma Aldrich, 5 wt% in a low-molecular-weight aliphatic alcohol and water) were purchased from Sigma-Aldrich. Phosphate-buffered saline (PBS) tablets were purchased from Gibco.

### Preparation of the TM-SnO_2_ films

Silicon wafers (10 mm × 10 mm) were washed with ethanol, acetone, and distilled water for several times. Then, they were sonicated and dried by N_2_ air followed by oxygen plasma treatment for 3 minutes. Each TM-SnO_2_ precursor solution was then spin coated onto respective silicon wafers at 2000 rpm for 30 seconds. Subsequently, the spin-coated films were annealed at 800 °C for 5 hours (heating rate of 5 °C/min) in ambient pressure.

### Preparation of the TM-SnO_2_

Nanoparticles were prepared by annealing each of the precursor solutions directly. Precursor solutions were put into Al_2_O_3_ crucibles, and then annealed at 800 °C for 5 hours using the same annealing procedure as used for the film synthesis.

### Fabrication of TM-SnO_2_-Nafion-modified GCE and electrochemical measurements of Cys oxidation

The electrochemical oxidation of Cys was investigated using glassy carbon electrodes (GCEs) modified with TM-SnO_2_ nanoparticles. For each M, a mass of 4.0 mg of TM-SnO_2_ nanoparticles was dispersed into 1.0 ml of distilled water containing 50 μl Nafion, and then mixed by using an ultrasonic processor (Wise cleaner, DAIHAN Sci., Wonju, Korea) for 10 minutes to obtain the homogeneous TM-SnO_2_-Nafion mixture. After that, a volume of 20 μl of the mixture was placed on a GCE, and was dried at 75 °C in a pre-heated oven for 30 minutes. A cyclic voltammogram (CV) of 10 mM Cys in PBS was obtained for each TM-SnO_2_-Nafion modified GCE.

### Characterizations

The morphologies of the samples were characterized by performing TEM (FEI Tecnai G^2^ F30 S-Twin) at an acceleration voltage of 300 kV. XRD patterns of the TM-SnO_2_ nanoparticles were obtained using Ni-filtered Cu–K*α* radiation from a Rigaku D/Max-A diffractometer. STXM was performed at the 10 A beamline at the Pohang Accelerator Laboratory (PAL). A Fresnel zone plate with an outermost zone width of 25 nm was used to focus the X-rays onto the TM-SnO_2_ nanoparticles on the TEM grids. Image stacks were acquired using X-ray absorption spectroscopy (XAS) to extract the transition metal *L*-edge, Sn *M*-edge, and O *K*-edge spectra. HRPES experiments were performed at the 8A1 beamline at PAL with an electron analyzer (Physical Electronics, PHI-3057). The binding energies of the core level spectra were determined with respect to the binding energy (E_B_ = 84.0 eV) of the clean Au 4 *f* core level for the same photon energy. The electrochemical experiments were performed by using a CHI617B potentiostat (CH Instruments, Austin, TX) with a three-electrode cell placed in a Faraday cage. A GCE with a diameter of 2 mm was used as the working electrode, a Pt wire with a diameter of 0.5 mm was used as the counter electrode, and the reference electrode was Ag/AgCl (3 M KCl). All working electrodes were polished with alumina (0.05 μm) paste on micro-cloth pads (Buehler, Lake Bluff, IL) prior to use. After measurements of electrochemical reaction of Cys with TM-SnO_2_-Nafion modified GCE, the catalytic activities were compared. The current values at 0.7 V (because of new small shoulder current) were selected to compare the catalytic activities of TM-SnO_2_ nanoparticles.

## Electronic supplementary material

Supplementary Information
