# Supplementary material for: Transition metal doped Sb@SnO2 nanoparticles for photochemical and electrochemical oxidation of cysteine
Source: Sci Rep. 2018 Aug 17;8:12348. doi: 10.1038/s41598-018-30962-0 (PMC6098097; doi:10.1038/s41598-018-30962-0)
Supplement: Supplementary file 1 — Supplementary Information [file 41598_2018_30962_MOESM1_ESM.docx]

**Supplementary Information**

**Transition metal doped Sb@SnO_2_ nanoparticles for photochemical and electrochemical oxidation of cysteine**

Yeonwoo Kim^1^, Sena Yang^2^, Yeji Kang^3^, Byung-Kwon Kim^3,*^, Hangil Lee ^3,*^

^1^ Molecular-Level Interfaces Research Center, Department of Chemistry, KAIST, Daejeon 34141, Republic of Korea

^2^ Center for Nano Characterization, Korea Research Institute of Standards and Science, Daejeon 305-400, Republic of Korea

^3^ Department of Chemistry, Sookmyung Women's University, Seoul 04310, Republic of Korea

*Corresponding Author

Hangil Lee Tel.: +82 2 710 9409; Fax: +82 2 2077 7321; E-mail: easyscan@sookmyung.ac.kr

Byung-Kwon Kim Tel.: +82 2 710 7808 Fax: +82 2 2077 7321; E-mail: kimbk@sookmyung.ac.kr

**ADDITIONAL INFORMATION**

**Figure S1.** The measurement of a sluggish oxidation current


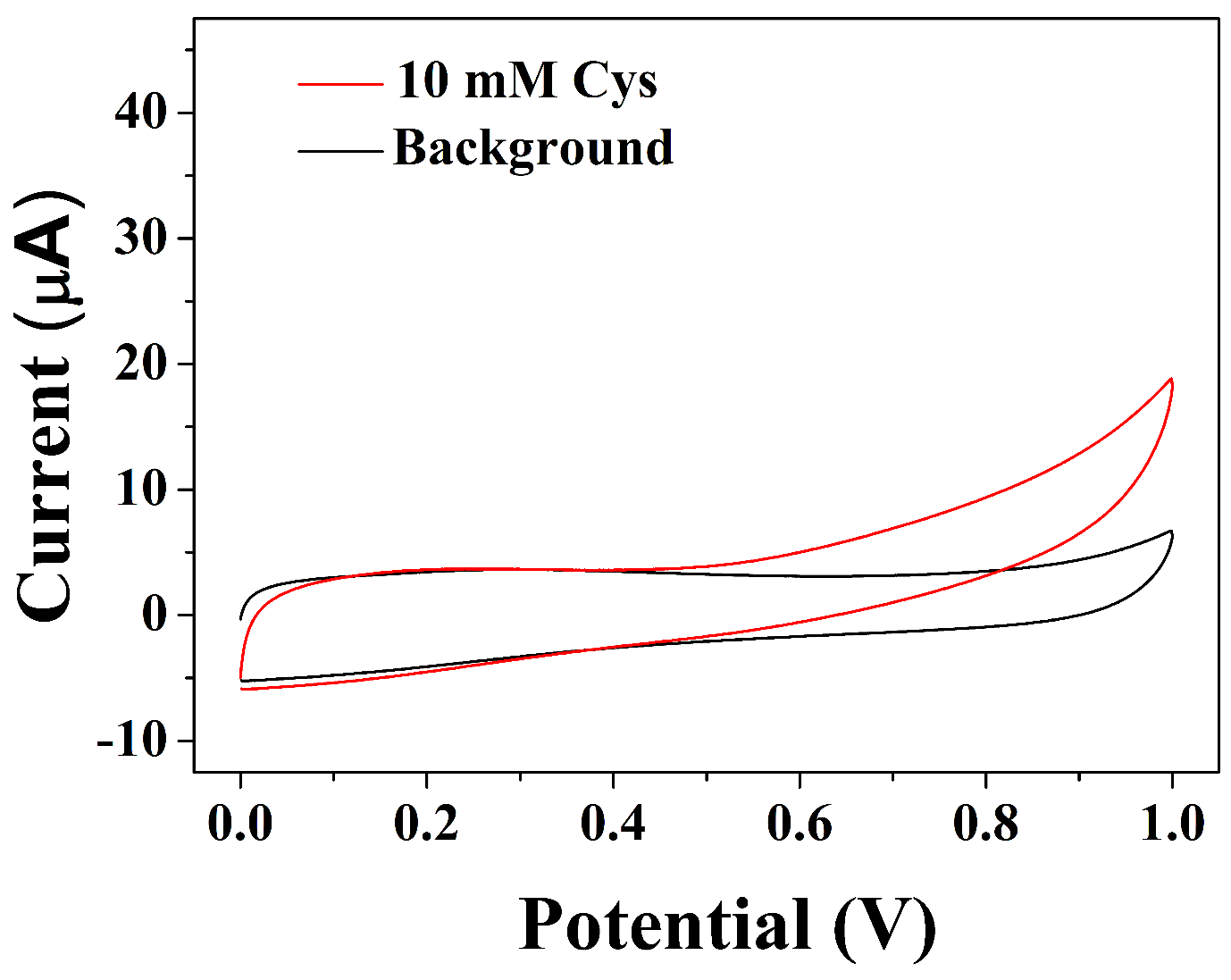


**Figure S1.** CVs obtained in PBS solution containing (red line) or not containing (black line)
10 mM Cys at a bare GCE. The scan rate was 50 mV/s.

As shown in Fig. S1, a sluggish oxidation current was observed at a bare GCE because of the intrinsically slow oxidation of Cys. To increase the current associated with the electrochemical oxidation of Cys, GCEs modified with catalytic TM@SnO_2_-Nafion were fabricated and used (see main text).

**Figure S2.** Conversion from CO to CO_2_


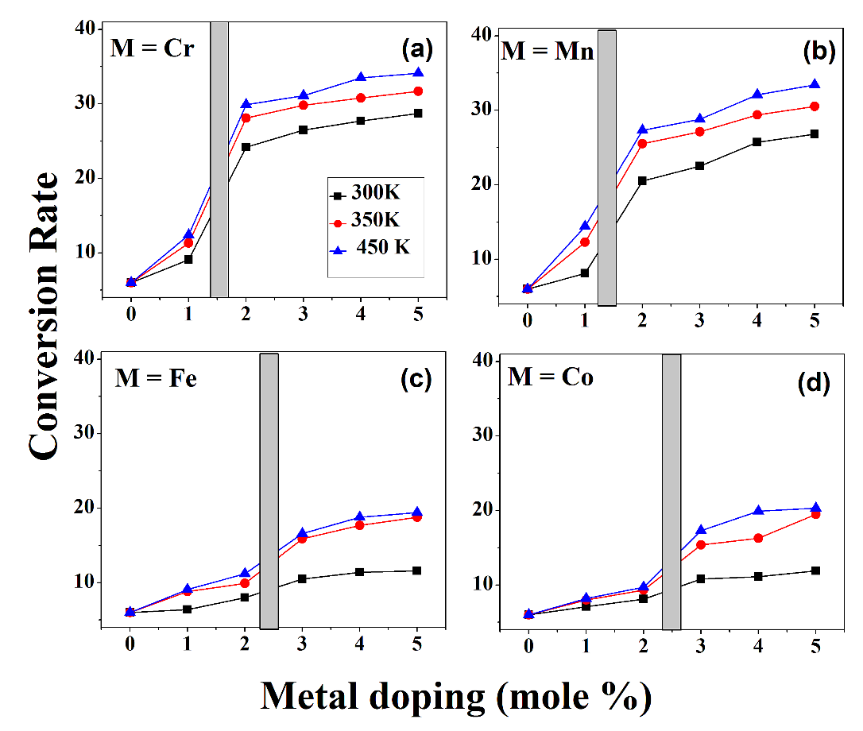


**Figure S2.** The rates of conversion of O_2_ and CO gas (1 × 10^-6^ torr) at 300 K to CO_2_ gas for various amounts of doped metal and various substrate temperatures in the presence of (a) Cr-SnO_2_, (b) Mn-SnO_2_, (c) Fe-SnO_2_, and (d) Co-SnO_2_ grown on silicon substrates.

A standard test for catalysts is the oxidation of CO to CO_2_. Mass spectrometry was carried out under ultra-high vacuum conditions to detect the CO, O_2_, and CO_2_ gas intensities with a Hiden RC 301 (mass range ~300 amu) system operating in positive-ion mode. Hence, we monitored the oxidation of CO to CO_2_ in the presence of the four distinct TM-SnO_2_ by using mass spectrometry for various substrate temperatures in the range 300 K ~ 450 K under UV irradiation. Fig. S2(a)~(d) show the reduced gas spectra obtained after the exposure of CO and O_2_ at 300 K to Cr-SnO_2_, Mn-SnO_2_, Fe-SnO_2_, or Co-SnO_2_ for 30 minutes (after 15 scans). Clearly, the rate of conversion of CO to CO_2_ was greater for either Cr-SnO_2_ or Mn-SnO_2_ than for the others, as expected.

**Figure S3.** HRPES data (Cr-SnO_2_)

**Figure S3.** HRPES data of Cr-SnO_2,_ (Left) O 1*s* and Sn 3*d* by increase of the amount of Cr dopants: 1 mol% (top), 3 mol% (middle), 5 mol% (bottom) ; (Upper right) Cr 2*p*; (Lower right) valence band by increase of the amount of Cr dopants: 1 mol% (black), 3 mol% (red), 5 mol% (blue).

**Figure S4.** HRPES data (Mn-SnO_2_)

**Figure S4.** HRPES data of Mn-SnO_2,_ (Left) O 1*s* and Sn 3*d* by increase of the amount of Mn dopants: 1 mol% (top), 3 mol% (middle), 5 mol% (bottom) ; (Upper right) Mn 2*p*; (Lower right) valence band by increase of the amount of Cr dopants: 1 mol% (black), 3 mol% (red), 5 mol% (blue).

**Figure S5.** HRPES data (Fe-SnO_2_)

**Figure S5.** HRPES data of Fe-SnO_2,_ (Left) O 1*s* and Sn 3*d* by increase of the amount of Fe dopants: 1 mol% (top), 3 mol% (middle), 5 mol% (bottom) ; (Upper right) Fe 2p; (Lower right) valence band by increase of the amount of Fe dopants: 1 mol% (black), 3 mol% (red), 5 mol% (blue).

**Figure S6.** HRPES data (Co-SnO_2_)

**Figure S6.** HRPES data of Co-SnO_2,_ (Left) O 1*s* and Sn 3*d* by increase of the amount of Co dopants: 1 mol% (top), 3 mol% (middle), 5 mol% (bottom) ; (Upper right) Co 2*p*; (Lower right) valence band by increase of the amount of Co dopants: 1 mol% (black), 3 mol% (red), 5 mol% (blue).
